# Reconstruction the feedback regulation of amino acid metabolism to develop a non-auxotrophic l-threonine producing *Corynebacterium glutamicum*

**DOI:** 10.1186/s40643-024-00753-9

**Published:** 2024-04-26

**Authors:** Jianhang Liu, Jiao Liu, Jiajun Li, Xiaojia Zhao, Guannan Sun, Qianqian Qiao, Tuo Shi, Bin Che, Jiuzhou Chen, Qianqian Zhuang, Yu Wang, Jibin Sun, Deqiang Zhu, Ping Zheng

**Affiliations:** 1 State Key Laboratory of Biobased Material and Green Papermaking, Qilu University of Technology, Shandong Academy of Sciences, Jinan, 250353 China; 2 Key Laboratory of Engineering Biology for Low-carbon Manufacturing, Tianjin Institute of Industrial Biotechnology, Chinese Academy of Sciences, Tianjin, 300308 China; 3 National Center of Technology Innovation for Synthetic Biology, Tianjin, 300308 China; 4 Shandong Provincial Key Laboratory of Microbial Engineering, School of Bioengineering, Qilu University of Technology, Shandong Academy of Sciences, Jinan, 250353 China; 5 Shandong University of Traditional Chinese Medicine, Jinan, 250355 China

**Keywords:** l-Threonine, *Corynebacterium glutamicum*, By-product, Allosteric regulation, Transport engineering

## Abstract

l-Threonine is an important feed additive with the third largest market size among the amino acids produced by microbial fermentation. The GRAS (generally regarded as safe) industrial workhorse *Corynebacterium glutamicum* is an attractive chassis for l-threonine production. However, the present l-threonine production in *C. glutamicum* cannot meet the requirement of industrialization due to the relatively low production level of l-threonine and the accumulation of large amounts of by-products (such as l-lysine, l-isoleucine, and glycine). Herein, to enhance the l-threonine biosynthesis in *C. glutamicum*, releasing the aspartate kinase (LysC) and homoserine dehydrogenase (Hom) from feedback inhibition by l-lysine and l-threonine, respectively, and overexpressing four flux-control genes were performed. Next, to reduce the formation of by-products l-lysine and l-isoleucine without the cause of an auxotrophic phenotype, the feedback regulation of dihydrodipicolinate synthase (DapA) and threonine dehydratase (IlvA) was strengthened by replacing the native enzymes with heterologous analogues with more sensitive feedback inhibition by l-lysine and l-isoleucine, respectively. The resulting strain maintained the capability of synthesizing enough amounts of l-lysine and l-isoleucine for cell biomass formation but exhibited almost no extracellular accumulation of these two amino acids. To further enhance l-threonine production and reduce the by-product glycine, l-threonine exporter and homoserine kinase were overexpressed. Finally, the rationally engineered non-auxotrophic strain ZcglT9 produced 67.63 g/L (17.2% higher) l-threonine with a productivity of 1.20 g/L/h (108.0% higher) in fed-batch fermentation, along with significantly reduced by-product accumulation, representing the record for l-threonine production in *C. glutamicum*. In this study, we developed a strategy of reconstructing the feedback regulation of amino acid metabolism and successfully applied this strategy to *de novo* construct a non-auxotrophic l-threonine producing *C. glutamicum*. The main end by-products including l-lysine, l-isoleucine, and glycine were almost eliminated in fed-batch fermentation of the engineered *C. glutamicum* strain. This strategy can also be used for engineering producing strains for other amino acids and derivatives.

**Supplementary Information:**

The online version contains supplementary material available at 10.1186/s40643-024-00753-9.

## Introduction

l-Threonine is an essential amino acid for human and livestock, which is the second limiting amino acid in swine feeds besides l-lysine and the third limiting amino acid in poultry feeds besides l-lysine and l-methionine (Dong et al. 2011). Therefore, l-threonine has been widely used in food, animal feed, and medicine, and its global market size is over 700,000 tons per year (Wendisch 2020). It is predominantly produced through microbial fermentation. Until now, many efforts have been made to optimize the fermentative production of l-threonine to meet the growing demand.

*Corynebacterium glutamicum* is a GRAS (generally regarded as safe) and robust microorganism for large-scale fermentation. It has been widely used for the industrial production of amino acids, such as l-glutamate, l-lysine, l-arginine, and branched-chain amino acids (Lee et al. 2016). With metabolic engineering, *C. glutamicum* can produce over 200 g/L l-lysine in fed-batch fermentation (Xu et al. 2020). Since both l-threonine and l-lysine belong to the aspartic family of amino acids, *C. glutamicum* may be also a promising chassis for industrial production of l-threonine. However, compared with the engineered *Escherichia coli* strains capable of producing over 100 g/L l-threonine, the reported l-threonine production by *C. glutamicum* is not satisfactory since the highest titer was only 57.7 g/L (Ishida et al. 1994). A major challenge for l-threonine production by *C. glutamicum* is the accumulation of large amounts of by-products such as l-lysine, l-isoleucine, and glycine. For example, the reported l-threonine producing strain with the highest titer is auxotrophic for l-isoleucine and produced 5.2 g/L l-lysine after 100 h of fed-batch fermentation (Ishida et al. 1994), another strain obtained by overexpression of *hom*^*r*^*-thrB* operon in a l-lysine producing strain produced ∼ 8 g/L l-threonine along with ∼ 4 g/L l-isoleucine (Reinscheid et al. 1994). Reducing by-products formation is an effective approach to enhance the metabolic flux into target product and simplify downstream purification and recovery process. Knocking out genes involved in by-product metabolism process, weakening the expression or activity of key enzymes and overexpressing l-threonine exporter have been employed to eliminate or reduce by-product in l-threonine production, respectively (Diesveld et al. 2009; Dong et al. 2016; Lv et al. 2012; Shiio 1990; Simic et al. 2002; Wei et al. 2018). Reported modifications just partially reduced accumulation of by-products or often led to auxotrophic strains, which may increase the industrial manufacture cost and cause the control difficulties of fermentation process. Therefore, the industrial application potential of these strategies is very limited. Deleting the genes of *ddh* (encoding diaminopimelate dehydrogenase) and *lysE* (encoding l-lysine exporter) significantly reduced extracellular l-lysine (from 7.14 g/L to 0.42 g/L) in shake flasks, but substantially increased intracellular l-lysine (from < 0.25 g/L to > 1.00 g/L) (Dong et al. 2016). Deleting *ilvA* (encoding l-threonine dehydratase) or introducing its mutations with reduced enzyme activity resulted in l-isoleucine auxotrophic strains, which require supplementation with l-isoleucine in the fermentation medium (Diesveld et al. 2009; Wei et al. 2018). Overexpressing the genes responsible for the l-threonine export only indirectly helped to reduce by-products in part (Simic et al. 2002).

**Fig. 1:**
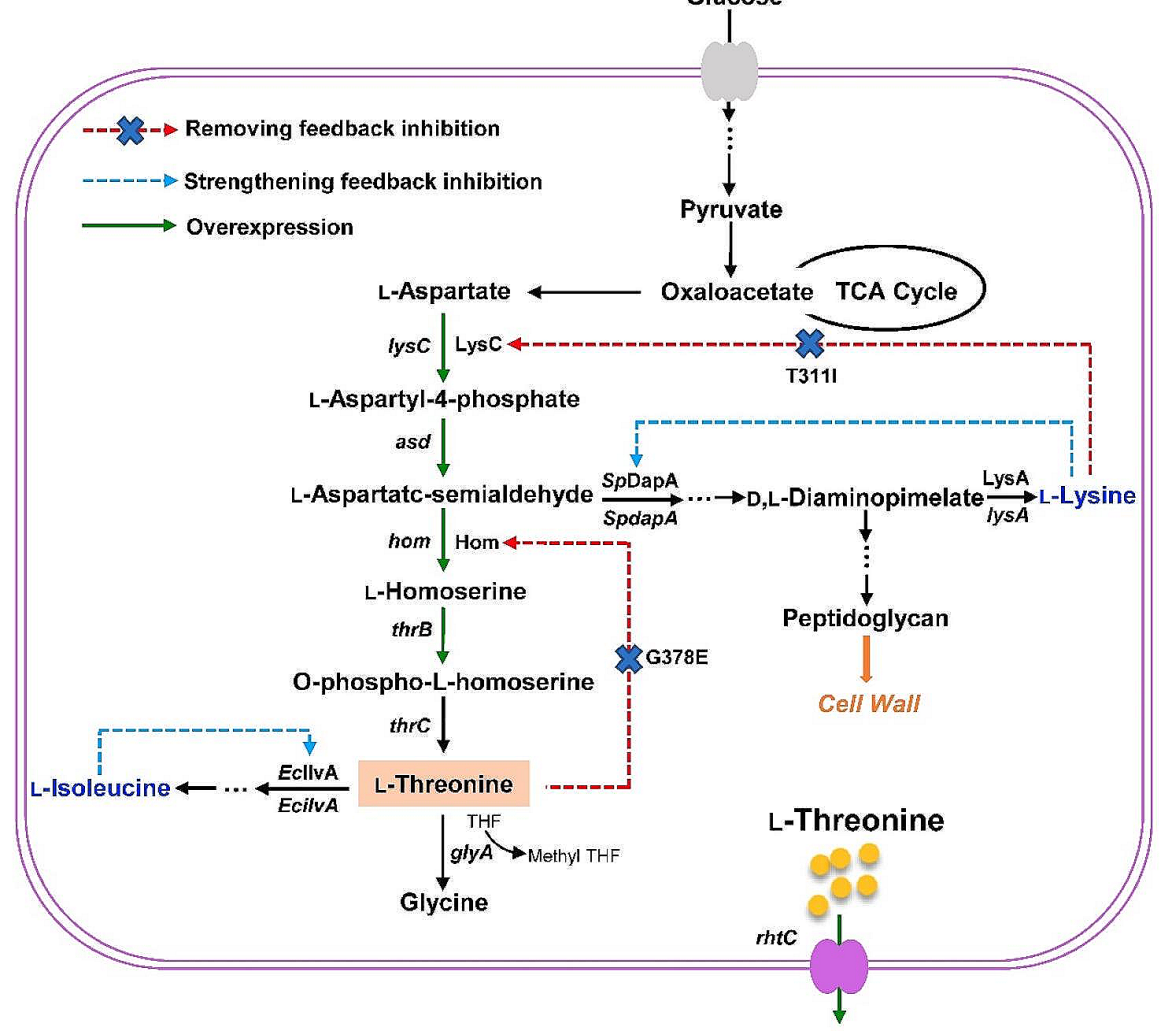
Construction of a non-auxotrophic l-threonine producing *C. glutamicum*. The point mutations T311I and G378E were employed to release LysC and Hom from feedback inhibition by l-lysine and l-threonine, respectively. The native genes were replaced with genes *SpdapA* and *EcilvA* encoding heterologous enzymes with strong feedback inhibition, respectively. LysC, aspartate kinase; Hom, homoserine dehydrogenase; SpDapA, dihydrodipicolinate synthase from *Streptococcus pneumoniae*; IlvA, threonine dehydratase from *E. coli*. THF, tetrahydrofolate; Methyl-THF, 5,10-methylene tetrahydrofolate

In this study, to construct an efficient l-threonine producing *C. glutamicum*, the l-threonine biosynthetic pathway was firstly enhanced by removal of the allosteric regulation of the key enzymes (LysC and Hom) and overexpression of multiple biosynthetic genes. Then, the feedback regulation of l-lysine and l-isoleucine biosynthetic pathways were strengthened by replacing the native enzymes with heterologous enzymes with more sensitive feedback inhibition. The l-threonine export gene *rhtC* from *E. coli* was overexpressed to further improve l-threonine production and decrease by-product accumulation (Fig. 1). The engineered strain displayed significantly improved l-threonine production and reduced by-products in fed-batch fermentation. The strategy of reconstructing the feedback regulation network presented here may be useful for enhancing the production of other amino acids and derivatives.

## Results

### Removal of feedback inhibition and overexpression of multiple key genes in the l-threonine biosynthesis pathway

**Fig. 2:**
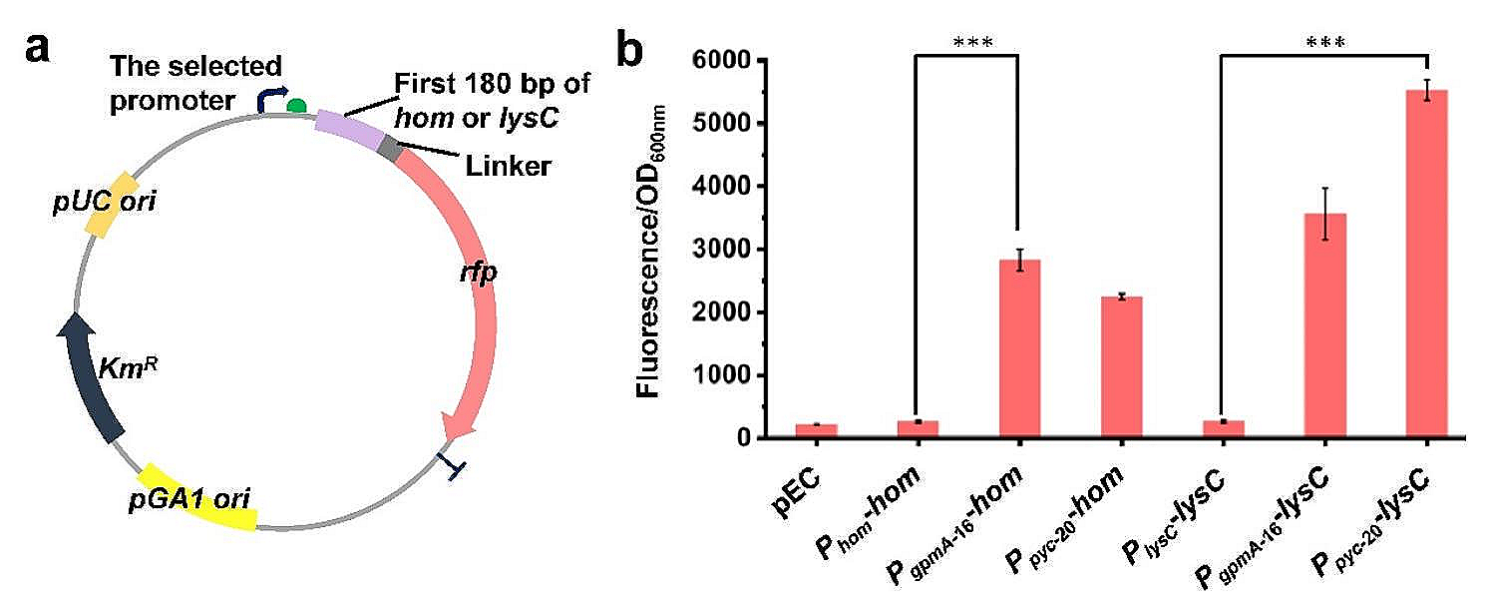
Selection of the strong promoters for overexpressing *lysC-asd* and *hom-thrB* operons. a A tailored reporter system for screening promoters for overexpressing *lysC-asd* and *hom-thrB* operons. The reporter gene was assembled by fusing the first 180 bp of target gene (*hom* or *lysC*) with a flexible linker (GGGGS)_3_ and a *rfp* gene. b Strength analysis of different promoters using the tailored reporter system. Data are presented as mean values +/− SD (*n* = 3 independent experiments). ***P < 0.001, student’s two-tailed *t*-test

In *C. glutamicum*, the l-threonine biosynthesis starts from l-aspartate, proceeding through the catalysis of five enzymes encoded by the genes *lysC*, *asd, hom*, *thrB*, and *thrC*, respectively (Fig. 1). Aspartate kinase (LysC) and homoserine dehydrogenase (Hom), as two key enzymes in the l-threonine synthesis pathway, are mainly subject to feedback inhibition by l-lysine and l-threonine, respectively. Therefore, removing the feedback inhibition of LysC and Hom is the first step for the development of l-threonine producers. Based on the previously published data (Ohnishi et al. 2002; Sahm et al. 1996), the frequently used mutants LysC^T311I^ and Hom^G378E^ were employed to release the feedback inhibition, forming the strain ZcglT1. Amino acid accumulation was analyzed in 24-deep-well plate cultivation. The final l-threonine concentration of 0.27 g/L was obtained after 24 h, and the same level of by-products (0.26 g/L l-lysine and 0.29 g/L glycine) were also accumulated in the medium (Fig. 2c).

In addition to the regulation at enzyme activity level, the expression of *lysC-asd* operon is repressed by l-threonine and l-isoleucine in *C. glutamicum*, and the *hom-thrB* operon is repressed by l-methionine (Li et al. 2017; Mateos et al. 1994). To remove the repression, constitutive strong promoters were used to enhance the expression of these genes. Two strong constitutive promoters (*P*_*gpmA−*16_ and *P*_*pyc−*20_) derived from the native promoters of *gpmA* and *pyc* genes, respectively, were further evaluated by using a tailored reporter system (Fig. 2a), which was previously reported to evaluate the fitness of promoter and target gene by our research group (Liu et al. 2022). Compared with the native promoter, *P*_*gpmA−*16_ and *P*_*pyc−*20_ led to 9.8- and 7.5-fold in the expression level of the *hom* reporter and 12.4- and 19.9-fold increases in that of the *lysC* reporter, respectively (Fig. 2b). To facilitate efficient expression, *P*_*gpmA−*16_ and *P*_*pyc−*20_ were selected to tune *hom*-*thrB* and *lysC*-*asd* operons, respectively. The same promoter shows different strengths for different target genes, which is mainly due to the influence of 5′ region of coding sequence (CDS) on gene expression (Mutalik et al. 2013; Tietze and Lale 2021).

**Fig. 3:**
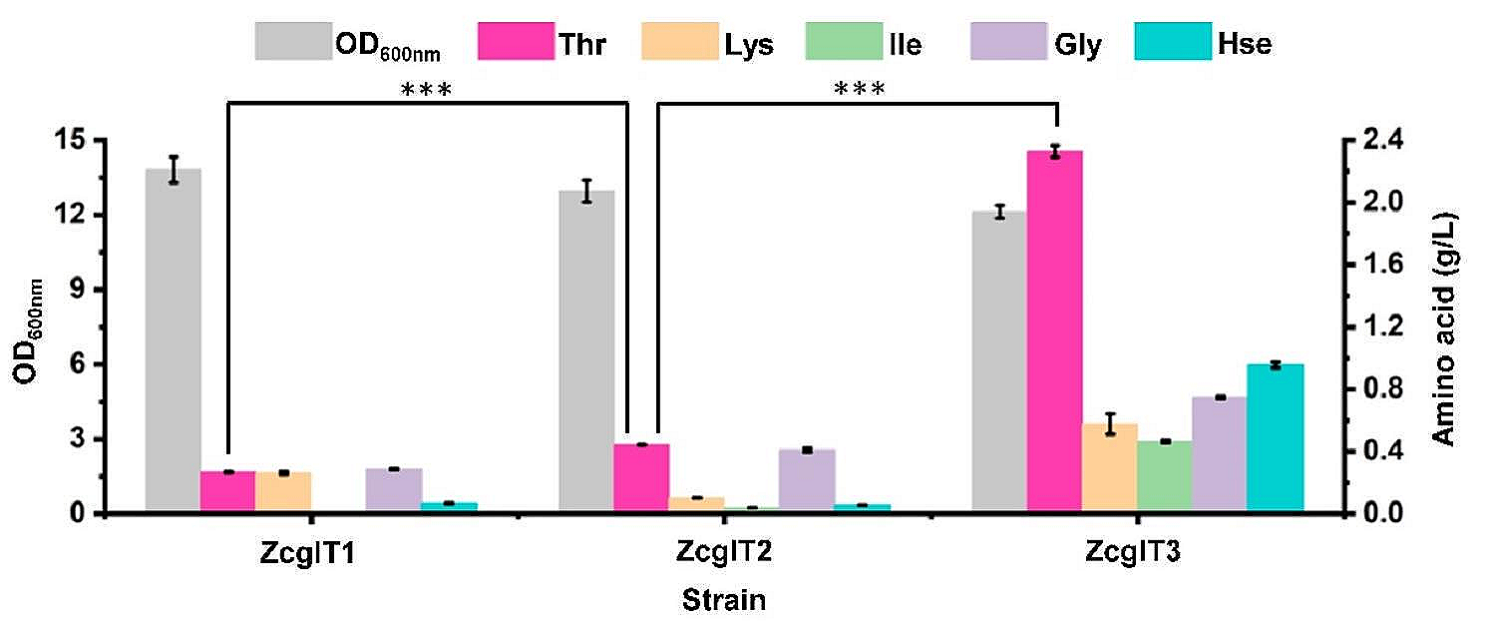
Removal of feedback inhibition and overexpression of multiple key genes for improving l-threonine production. The 24-deep-well plate fermentation of engineered strains at 30 °C for 24 h. ZcglT1, *C. glutamicum* ATCC 13,032 derivative with T311I mutantion of *lysC* and G378E mutantion of *hom*; ZcglT2, ZcglT1 derivative with insertion of a constitutive promoter *P*_*gpmA*−16_ in front of *hom*^G378E^; ZcglT3, ZcglT2 derivative with insertion of a constitutive promoter *P*_*pyc*−20_ in front of *lysC*^T311I^. Thr, l-threonine; Lys, l-lysine; Ile, l-isoleucine; Gly, glycine; Hse, l-homoserine. Data are presented as mean values +/− SD (*n* = 3 independent experiments). ***P < 0.001, student’s two-tailed *t*-test

Next, the two operons were tuned with the selected promoter variants. All the genetic modifications were carried out in the *C. glutamicum* chromosome. The promoter *P*_*gpmA*−16_ was *in-situ* inserted in front of the start codon of *hom*^G378E^ gene for expression enhancement of the *hom*^G378E^*-thrB* operon, resulting in strain ZcglT2. Insertion of *P*_*gpmA*−16_ promoter led to a 65.9% increase in l-threonine production (from 0.27 g/L to 0.44 g/L) in 24-deep-well plate cultivation (Fig. 3). Subsequently, the promoter *P*_*pyc*−20_ was *in-situ* inserted in front of the start codon of *lysC*^T311I^ gene to enhance the expression of the *lysC*^T311I^*-asd* operon. The resultant strain ZcglT3 produced 2.33 g/L l-threonine, which was 425.8% higher than that of its parent strain ZcglT2. Besides the target product, 0.58 g/L l-lysine, 0.47 g/L l-isoleucine, 0.75 g/L glycine, and 0.96 g/L l-homoserine were also detected in medium. The l-threonine producing mutant ZcglT3 showed a slight decrease in biomass compared with the strain ZcglT1 (12.2%), suggesting a redirection of metabolic flux from the production of biomass to the biosynthesis of the target product due to enhancement of the biosynthetic pathway.

### Strengthening allosteric regulation of dihydrodipicolinate synthase to reduce the by-product l-lysine

**Fig. 4:**
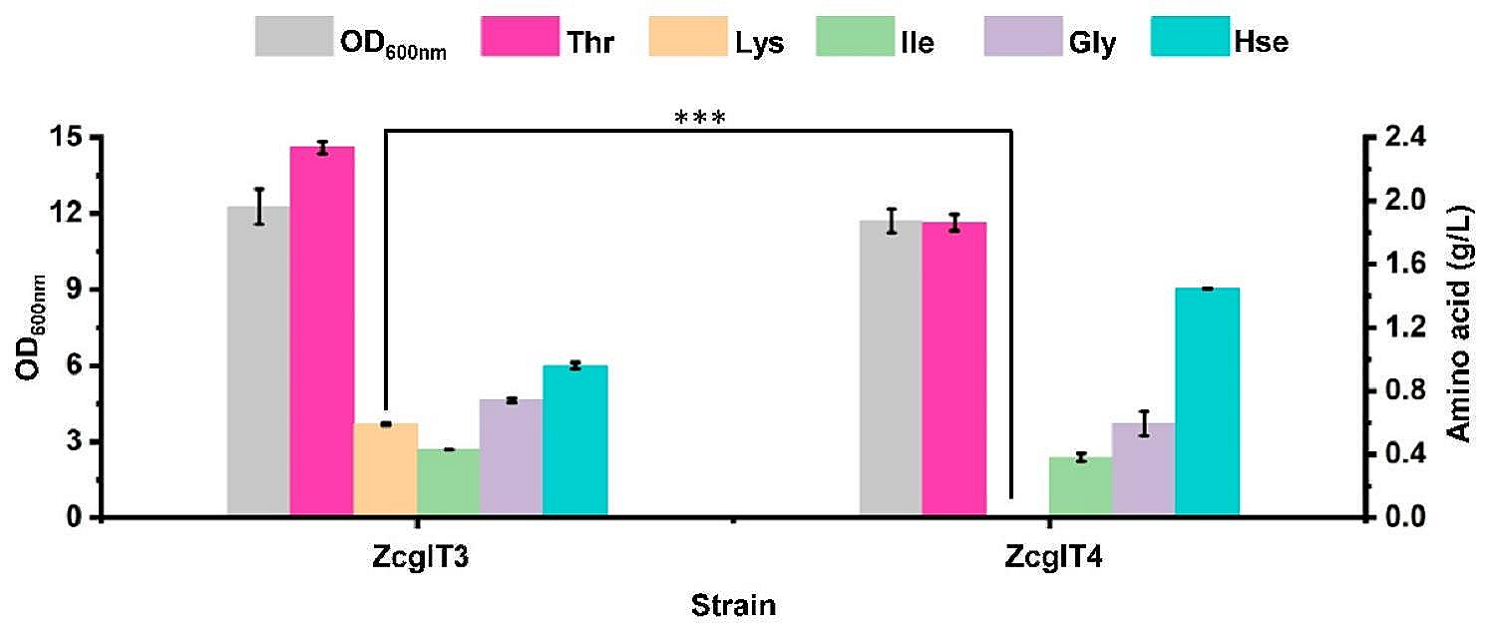
Replacing the native *CgdapA* gene with *SpdapA* in ZcglT3 to reduce the by-product l-lysine. The 24-deep-well plate fermentation was performed at 30 °C for 24 h. ZcglT4, ZcglT3 derivative with replacement of the native *CgdapA* with *SpdapA*. Thr, l-threonine; Lys, l-lysine; Ile, l-isoleucine; Gly, glycine; Hse, l-homoserine. Data are presented as mean values +/− SD (*n* = 3 independent experiments). ***P < 0.001, student’s two-tailed *t*-test

Chromosomally transcriptional overexpression of flux-control genes improved l-threonine production by nearly 8-fold. However, there was a large accumulation of by-products (e.g., 0.58 g/L l-lysine for ZcglT3) in culture medium. The main reason for l-lysine accumulation is that the key enzyme for l-lysine biosynthesis in *C. glutamicum*, dihydrodipicolinate synthase (DapA, EC 4.3.3.7), is free of feedback regulation by l-lysine (Geng et al. 2013). As a result, when the *lysC*^T311I^*-asd* operon was overexpressed in *C. glutamicum*, l-lysine was overproduced. Although blocking the synthesis pathway is expected to eliminate the formation of this by-product, a d,l-diaminopimelate (DAP) auxotrophic phenotype will be generated (Shiio 1990), which will require the supplementation of DAP for l-lysine formation and cell wall synthesis during fermentation (Fig. 1). In order to decrease the metabolic flux towards l-lysine biosynthesis and avoid auxotrophy, the heterologous DapA strictly inhibited by l-lysine was employed to strengthen the l-lysine feedback regulation in *C. glutamicum.* Because the DapA from *Streptococcus pneumoniae* (*Sp*DapA) displays an IC_50_ of 0.06 mM l-lysine (Soares Da Costa et al. 2016), the *SpdapA* gene was synthesized and used to replace the native *CgdapA* gene in strain ZcglT3, resulting in strain ZcglT4. As expected, this modification completely eliminated the accumulation of l-lysine in strain ZcglT4 after 24 h of deep-well plate cultivation (Fig. 4). There was no obvious difference (*P* > 0.05) in cell growth after the replacement of *SpdapA*, suggesting that strain ZcglT4 could synthesize sufficient l-lysine for cell growth. Unexpectedly, the strain ZcglT4 only accumulated 1.86 g/L l-threonine, which was 20.3% lower than the parental strain ZcglT3 (2.34 g/L). Meanwhile, the strain ZcglT4 accumulated 1.45 g/L l-homoserine, which was 50.8% higher than the parental strain ZcglT3 (0.96 g/L). Hence, we supposed that the conversion of l-homoserine to l-threonine becomes likely a new bottleneck for l-threonine production in strain ZcglT4. Taken together, these results suggest that the enhancement of l-lysine feedback regulation is an effective strategy for down-regulation of the competitive l-lysine branch pathway for l-threonine production.

### Strengthening allosteric regulation of l-threonine dehydratase to reduce the by-product l-isoleucine

**Fig. 5:**
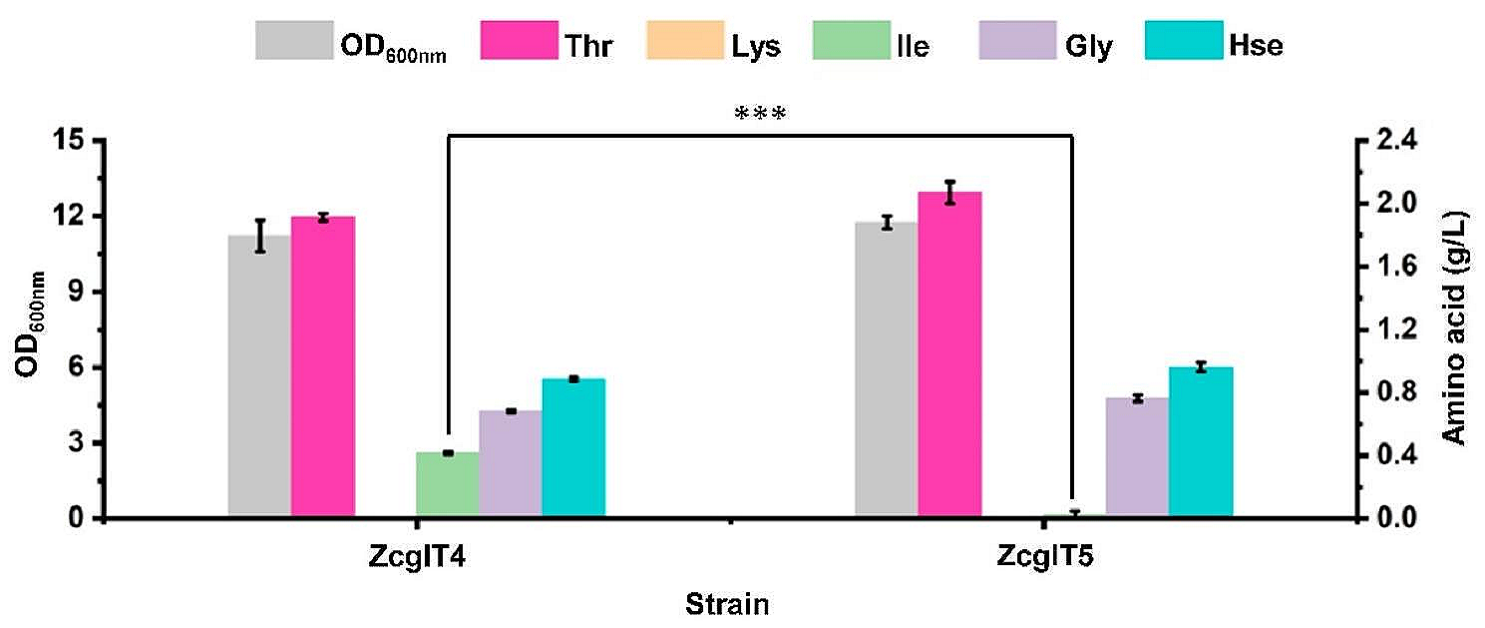
Replacing the native *CgilvA* gene with *EcilvA* in ZcglT4 to reduce the by-product l-isoleucine. The 24-deep-well plate fermentation of engineered strains was performed at 30 °C for 24 h. ZcglT5, ZcglT4 derivative with replacement of the native *CgilvA* with *EcilvA*. Thr, l-threonine; Lys, l-lysine; Ile, l-isoleucine; Gly, glycine; Hse, l-homoserine. Data are presented as mean values +/− SD (*n* = 3 independent experiments). ***P < 0.001, student’s two-tailed *t*-test

l-Isoleucine is another main by-product for l-threonine production in *C. glutamicum* (Guillouet et al. 2001; Morbach et al. 1995). Strain ZcglT4 accumulated 0.38 g/L l-isoleucine after 24 h of deep-well plate cultivation. The conversion of l-threonine to l-isoleucine is initiated by the l-threonine dehydratase (IlvA, EC 4.2.1.16) encoded by *ilvA* gene. The IlvA of *C. glutamicum* (*Cg*IlvA) is weakly inhibited by l-isoleucine, with an approximate IC_50_ of 1 mM (Guo et al. 2015; Hou et al. 2012). As a result, enhancement of the l-threonine biosynthesis led to substantial accumulation of l-isoleucine. However, the IlvA of *E. coli* (*Ec*IlvA) exhibits a strong inhibition with a much lower IC_50_ of 0.067 mM l-isoleucine (Wu et al. 2021), which is a perfect choice for enhancing the feedback regulation of l-isoleucine biosynthesis in *C. glutamicum*. To reduce the conversion of l-threonine to l-isoleucine, the *EcilvA* gene was used to replace the native *CgilvA* gene in strain ZcglT4, generating strain ZcglT5. The accumulated l-isoleucine of strain ZcglT5 (0.03 g/L) dramatically reduced by 93.7% compared with the parental strain ZcglT4 (0.42 g/L) (Fig. 5). Simultaneously, the l-threonine produced by strain ZcglT5 was slightly increased by 8.1% in comparison to the parental strain. The production of cell biomass and other amino acids were not significantly affected. These results show that strengthening feedback regulation of l-isoleucine synthesis by using heterologous IlvA with strong allosteric inhibition benefits the elimination of by-product l-isoleucine and does not impair cell growth and l-threonine production.

### Transport engineering and overexpression of homoserine kinase to further enhance l-threonine production and reduce by-products

Engineering allosteric regulation of key enzymes has dramatically reduced the accumulation of by-products l-lysine and l-isoleucine. However, l-threonine production remained low, and the by-products glycine (0.76 g/L) generated by degradation of l-threonine and the intermediate metabolite l-homoserine (0.96 g/L) were still accumulated in the fermentation broth (Fig. 5). Transport engineering is an important strategy to promote amino acid excretion and improve its production, which is also helpful to reduce degradation of the target molecule and accelerate the conversion of intermediate metabolite. Several l-threonine exporters have been reported, including ThrE and SerE from *C. glutamicum* and RhtA, RhtB, and RhtC from *E. coli*. However, these exporters are not specific for transporting l-threonine and can also mediate the excretion of other amino acids such as l-serine, l-homoserine, and l-proline (Diesveld et al. 2009; Simic et al. 2001; Zakataeva et al. 1999; Zhang et al. 2020). Diesveld et al. tested the effects of overexpressing RhtA, RhtB, and RhtC on l- threonine production in *C. glutamicum*, and RhtC showed the largest improvement in l- threonine titer (Diesveld et al. 2009). Endogenous ThrE has been used to increase l-threonine production in *C. glutamicum*, but SerE has not been tested. Therefore, we cloned the *thrE*, *serE*, and *rhtC* genes and constructed three overexpression plasmids, where the exporter gene was driven by IPTG-induced promoter *P*_*trc*_.

**Fig. 6:**
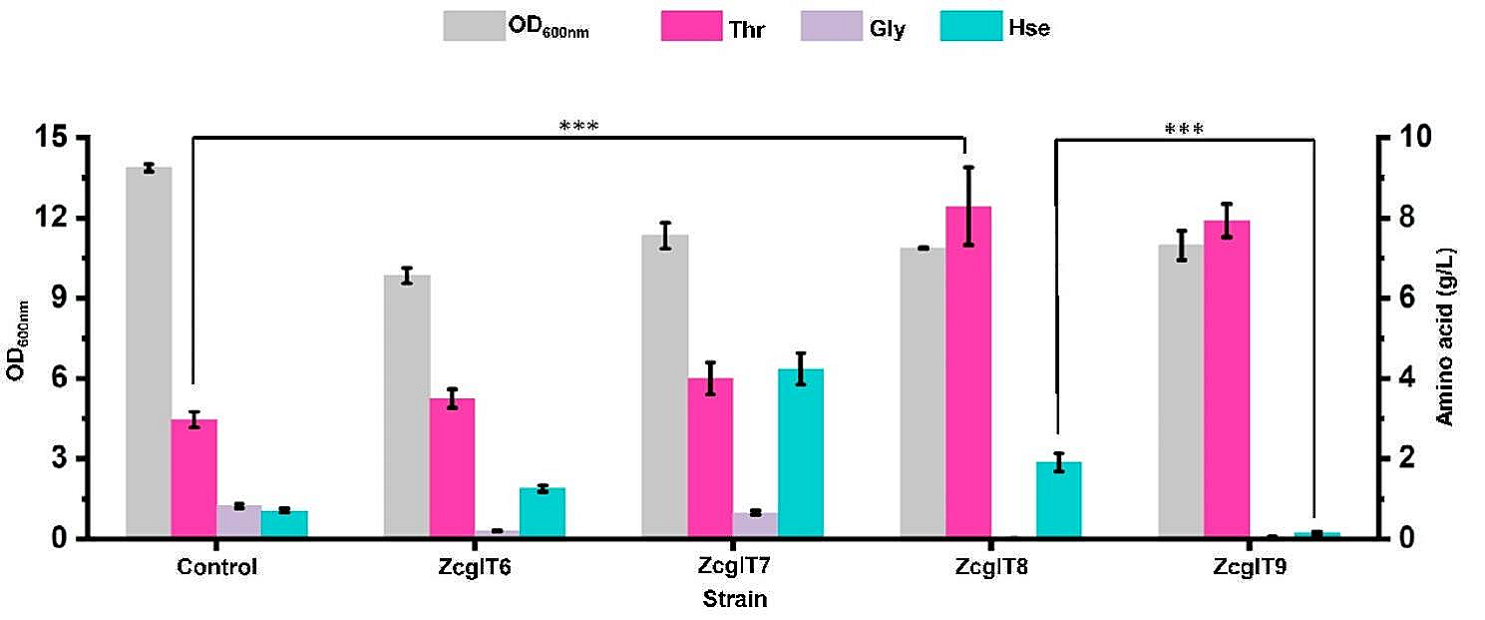
Overexpression of l-threonine exporter and homoserine kinase to enhance l-threonine production and reduce by-product accumulation. The 24-deep-well plate fermentation was performed at 30 °C for 36 h with 0.5 mM IPTG. Control, ZcglT5 derivative harboring pEC-XK99E empty plasmid; ZcglT6, ZcglT5 derivative harboring pEC-*thrE* plasmid; ZcglT7, ZcglT5 derivative harboring pEC-*serE* plasmid; ZcglT8, ZcglT5 derivative harboring pEC-*rhtC* plasmid; ZcglT9, ZcglT5 derivative harboring pEC-*rhtCthrB* plasmid. Thr, l-threonine; Lys, l-lysine; Ile, l-isoleucine; Gly, glycine; Hse, l-homoserine. Data are presented as mean values +/− SD (*n* = 3 independent experiments). ***P < 0.001, student’s two-tailed *t*-test

The overexpression plasmids with *thrE*, *serE*, and *rhtC* genes were transferred into the strain ZcglT5, resulting in strains ZcglT6, ZcglT7, and ZcglT8, respectively. The resulting strains and the control strain (ZcglT5 with an empty plasmid) were cultivated in 24-deep-well plate at 30 °C for 36 h with 0.5 mM IPTG. All strains with exporter overexpression resulted in increased l-threonine production and a small decrease in cell growth (Fig. 6). Strains ZcglT6, ZcglT7, and ZcglT8 produced 3.50 g/L, 4.00 g/L, and 8.29 g/L l-threonine, which increased by 17.6%, 34.3%, and 178.7% compared with the control strain (2.98 g/L), respectively. We also measured the intracellular l-threonine concentrations of these strains, and found that strains ZcglT6 and ZcglT8 decreased by 26.5% and 39.6% compared with the control strain (143.7 mM), respectively, while strain ZcglT7 showed no significant decrease (Fig. S1). Moreover, Strains ZcglT6, ZcglT7, and ZcglT8 produced 0.20 g/L, 0.66 g/L, and 0.02 g/L glycine, which decreased by 75.4%, 20.2%, and 97.4% compared with the control strain (0.82 g/L), respectively. The accumulation of l-lysine and l-leucine was still very low in all the engineered strains. Due to the highest l-threonine production and the lowest glycine accumulation, RhtC was more beneficial to efficient production of l-threonine. Meanwhile, overexpression of all three exporters gave rise to increased l-homoserine accumulation. This could be caused by the function of overexpressed exporters, since previous studies have shown that RhtA and RhtB can export l-homoserine (Livshits et al. 2003; Zakataeva et al. 1999). We speculate that ThrE, SerE, and RhtC may also export l-homoserine.

To promote the conversion of l-homoserine to l-threonine in vivo and reduce l-homoserine accumulation, *thrB* gene encoding homoserine kinase (ThrB) was further overexpressed under the control of a strong constitutive promoter on the plasmid with *rhtC* gene, generating strain ZcglT9. After 36 h of 24-deep-well plate cultivation, l-homoserine was significantly reduced to 0.15 g/L in strain ZcglT9, which was 92.3% lower than that of strain ZcglT8 (1.91 g/L). There is no difference in l-threonine production and accumulation of other by-products (l-lysine, l-isoleucine, and glycine) between strains ZcglT9 and ZcglT8. These results suggest that co-expression of *rhtC* and *thrB* genes can exert a strong driven force to significantly increase l-threonine production and reduce the accumulation of glycine and l-homoserine.

### l-Threonine production in fed‑batch fermentation

**Fig. 7:**
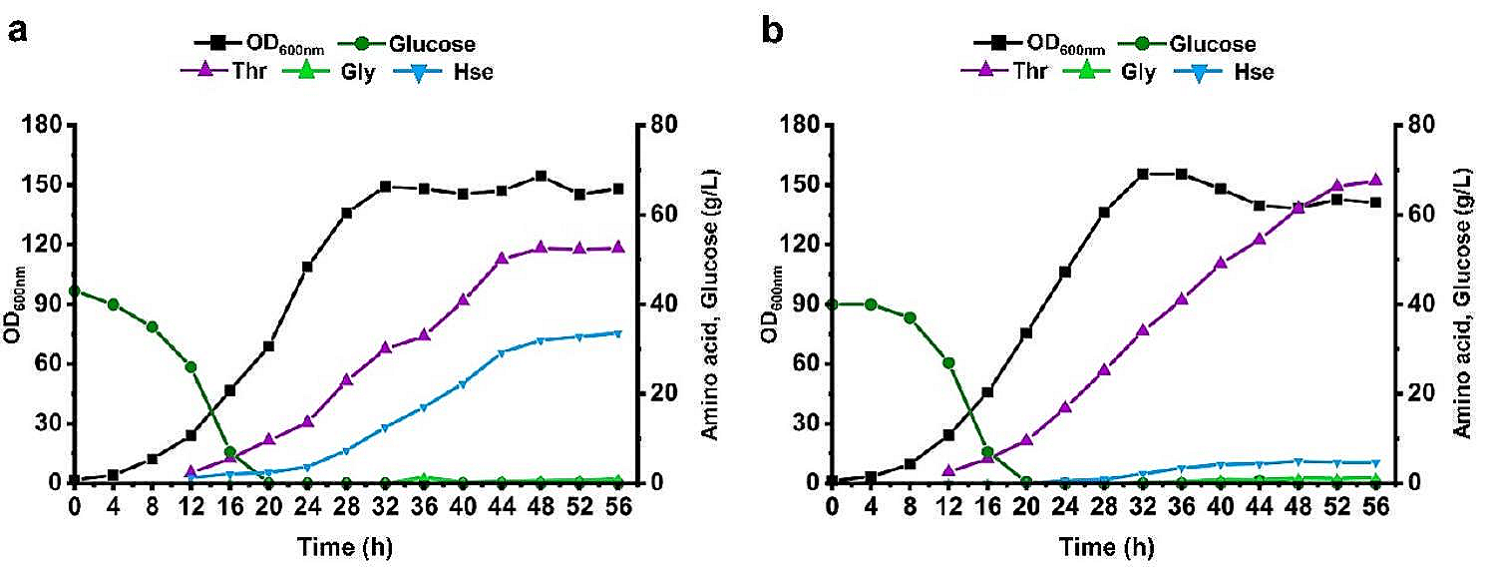
Fed-batch fermentation of strains ZcglT8 and ZcglT9 in 5 L bioreactors. **a** Strain ZcglT8, **b** Strain ZcglT9. Amino acids were detected from 12 h. Thr, l-threonine; Lys, l-lysine; Ile, l-isoleucine; Gly, glycine; Hse, l-homoserine

To further evaluate the l-threonine-producing capacity of the engineered strains, the fed-batch fermentation of the best two strains ZcglT8 and ZcglT9 was performed in 5 L bioreactors. Since the engineered strains were non-auxotrophic, amino acids were not exogenously added in the fermentation medium. Both strains exhibited similar growth patterns and the maximum OD_600nm_ values reached approximately 150 at 32 h (Fig. 7). A rapid increase in l-threonine production was observed when the initial glucose (50 g/L) in the medium was consumed and the feeding was started (about 20 h). After 56 h fermentation, the strain ZcglT8 produced 52.52 g/L l-threonine with a yield of 0.16 g/g glucose and a productivity of 0.93 g/L/h. For comparison, the strain ZcglT9 produced 67.63 g/L l-threonine with a yield of 0.21 g/g glucose and a productivity of 1.20 g/L/h under the same test condition. The titer, productivity, and yield of strain ZcglT9 were improved by 28.8%, 31.3%, and 29.0%, respectively, compared with strain ZcglT8. Notably, the byproduct l-homoserine of strain ZcglT9 was largely decreased to 4.58 g/L, which was only 13.7% of the l-homoserine produced by strain ZcglT8 (33.52 g/L). For both strains, no l-lysine was accumulated in the fermentation process and only small amounts of l-isoleucine and glycine (∼ 1 g/L) were detected in the final fermentation broth. Compared with the 24-deep-well plate fermentation, the accumulation of l-homoserine was increased in the fed-batch fermentation in 5 L bioreactors, which might be due to the stronger production intensity and longer cultivation time. Further effort is needed to promote the metabolism from l-homoserine to l-threonine or block the efflux of l-homoserine by engineering specific transporter for l-threonine.

## Discussion

As an essential amino acid for human and animal nutrition, l-threonine has extensive applications in food, medicine, and feed. Because of the biosafety in industrial production and the robustness of metabolic regulation and stress tolerance, *C. glutamicum* has played a leading role in amino acid production (Tsuge et al. 2021, Wendisch 2020). However, limited production level and the accumulation of various by-products, especially end by-products including l-lysine, l-isoleucine, and glycine, are main bottlenecks for l-threonine production with *C. glutamicum*. So far, the highest level of l-threonine production in the *C. glutamicum* was achieved by an l-isoleucine auxotroph, which produced 57.7 g/L l-threonine in 100 h with a productivity of 0.577 g/L/h and 5.7 g/L l-lysine (Ishida et al. 1994). In comparison, the final strain ZcglT9 reconstructed in this study exhibited an improvement of 17.2% in l-threonine titer (67.63 g/L) in 56 h with an increase of 108.0% for productivity (1.20 g/L/h), and it was a non-auxotrophic l-threonine producer. Moreover, strain ZcglT9 no longer produced l-lysine and only accumulated minor l-isoleucine and glycine (∼ 1 g/L) in fed-batch fermentation, but some l-homoserine (4.58 g/L) was still generated (Fig. 6). The possible reason for l-homoserine accumulation is that ThrB is feedback inhibited by l-threonine in a competitive inhibitory mechanism with a *K*_*i*_ of 4.73 mM and l-threonine exporter RhtC also transported l-homoserine (Fig. 5) (Petit et al. 2018). Therefore, ThrB variant with released sensitivity to feedback inhibition by l-threonine and l-threonine specific exporter are hopeful to further reduce l-homoserine accumulation.

Allosteric regulation of key enzymes plays important roles in the dynamic control of amino acid metabolism (Lang et al. 2014; Sander et al. 2019). Usually, removing feedback inhibition of synthetic pathway is required for efficient amino acid production. However, strengthening allosteric regulation has not been reported for the reduction of amino acid by-product. DapA and IlvA, as key enzymes of the l-lysine and l-isoleucine pathway respectively, were applied to successfully decrease by-product by the replacement of heterologous strong feedback-inhibited enzymes in *C. glutamicum*l-threonine producer (Figs. 3 and 4). No l-lysine was accumulated in engineered strains with *SpdapA* gene encoding l-lysine-sensitive *Sp*DapA instead of the native *CgdapA*, and no need to add l-lysine in fermentation medium (Fig. 6). The *dapA* mutants and a double knockout mutant (*ddh* and *lysE*) were previously reported to reduce l-lysine synthesis in l-threonine production, but which led to the limited reduction of l-lysine (Dong et al. 2016; Lv et al. 2012; Shiio 1990). l-Lysine is a common by-product for the production of l-methionine, l-homoserine, l-isoleucine, and other products synthesized from l-aspartate-4-semialdehyde in *C. glutamicum*. Gene expression knockdown of *dapA* by changing the start codon ATG to GTG or attenuating its promoter is often employed to lessen l-lysine accumulation in their production, but it is still formed (Li et al. 2016, 2021; Vogt et al. 2015). The strategy for engineering *dapA* gene in this study should also be used for strain modification of these products. It has been proved that similar replacement for *ilvA* gene could significantly reduce l-isoleucine accumulation. The known mutants displayed stronger feedback inhibition probably facilitate no l-isoleucine at all (Chen et al. 2013).

## Conclusions

In summary, we developed a highly efficient strategy for by-product control of *C. glutamicum*l-threonine producer, which dramatically reduced by-product accumulation but didn’t lead to aminophenol auxotroph. In combination with modification of synthetic pathway and transport engineering, a genetically defined l-threonine producing strain were successfully constructed and exhibited higher titer (67.63 g/L) and productivity (1.20 g/L/h) than that of the previously reported strains in a 5 L fed-batch fermentation. The resulting strain exhibited an encouraging starting strain for the industrial production of l-threonine with *C. glutamicum*. Further genetic alterations to regulate the metabolic flux for l-threonine and reduce the efflux of l-homoserine are suggested to generate a more promising l-threonine producer.

## Materials and methods

### Strains and culture conditions

All strains constructed in this study are listed in Table 1. *E. coli* strain Trans1-T1 used for plasmid cloning was cultivated aerobically at 37 °C in Luria-Bertani (LB) medium. Kanamycin (50 µg/mL) was added to the medium as required. *C. glutamicum* strains were cultivated aerobically at 30 °C in TSB medium containing 5 g/L glucose, 9 g/L soya peptone, 5 g/L yeast extract, 1 g/L K_2_HPO_4_·3H_2_O, 0.1 g/L MgSO_4_·7H_2_O, 3 g/L urea, 0.5 g/L succinic acid, 10 µg/L biotin, 100 µg/L vitamin B1, and 20 g/L MOPS (pH 7.2). Kanamycin (25 µg/mL) was added as required.

**Table 1:** Strains used in this study

Strain	Description	Reference or source^a^
E. coli		
Trans1-T1	General cloning host	TransGen Biotech
***C. glutamicum***		
ATCC 13,032	Wild-type strain	ATCC
ZcglT1	ATCC 13,032 derivative with T311I mutantion of *lysC* and G378E mutantion of *hom*	This study
ZcglT2	ZcglT1 derivative with insertion of a constitutive promoter *P*_*gpmA*−16_ in front of *hom*^G378E^	This study
ZcglT3	ZcglT2 derivative with insertion of a constitutive promoter *P*_*pyc*−20_ in front of *lysC*^T311I^	This study
ZcglT4	ZcglT3 derivative with replacement of the native *CgdapA* with *SpdapA*	This study
ZcglT5	ZcglT4 derivative with replacement of the native *CgilvA* with *EcilvA*	This study
ZcglT6	ZcglT5 derivative harboring pEC-*thrE* plasmid	This study
ZcglT7	ZcglT5 derivative harboring pEC-*serE* plasmid	This study
ZcglT8	ZcglT5 derivative harboring pEC-*rhtC* plasmid	This study
ZcglT9	ZcglT5 derivative harboring pEC-*rhtCthrB* plasmid	This study

^a^ ATCC: American Type Culture Collection

### Plasmid construction

Plasmids used in this study are listed in Table S1. Primers and details for constructing plasmids are described in Table S2. The sequences of promoter used in this study are listed in Table S3. Plasmids were constructed via recombination, which was performed using the ClonExpress MultiS One Step Cloning Kit (Vazyme, Nanjing, China). DNA polymerase and reagents used for PCR were purchased from TransGen Biotech (Beijing, China). Services of primer, gene synthesis, and DNA sequencing were provided by GENEWIZ Inc. (Suzhou, China).

Here, the plasmid construction of pK18-*hom*^G378E^ was presented as an example. To construct plasmid pK18-*hom*^G378E^, the left and right homologous fragments with mutation were amplified from the genomic DNA of *C. glutamicum* by PCR using primer pairs *hom-*B1/ *hom-*B2-378 and *hom*378-2/ *hom*-B3, respectively. The plasmid backbone was amplified from the empty plasmid pK18*mobsacB* by PCR using primer pair pK18-1/ pK18-2. Then these three DNA fragments were purified and ligated using the CloneExpress® MultiS One Step Cloning Kit (Vazyme Biotech, Nanjing, China) to generate the final plasmid. The construction process of other plasmids was similar to that of plasmid pK18-*hom*^G378E^.

### Genetic manipulation of *C. Glutamicum*

A pK18*mobsacB* derived plasmid that harbors two ∼ 500 bp homologous arms flanking at both sides of the mutation or target fragment was used to modify chromosome in *C. glutamicum*. For DNA modification in *C. glutamicum*, 1 µg plasmid (e.g., pK18-*hom*^G378E^) was transformed into *C. glutamicum* via electroporation, and mutant was obtained by two rounds of homologous recombination as described previously (Schäfer et al. 1994). Transformants were firstly plated on TSB solid medium supplemented with 25 µg/mL kanamycin for screening single-crossover mutants. Next, the resulting recombinants grew in TSB medium without kanamycin for 4 h, and the culture with an appropriate dilution (10^4^-10^5^ cells) was spread on TSB plates containing 10% sucrose for screening double-crossover mutants. The final mutants were verified by colony PCR and Sanger sequencing.

### Promoter evaluation

The promoter was ligated with the corresponding tailored RFP reporter gene and pEC-XK99E plasmid to construct the reporter plasmid. The plasmid was transformed into *C. glutamicum* via electroporation. For seed preparation, strains were cultivated in 24-deep-well plates containing 800 µL TSB medium in each well. The preculture was used to inoculate 800 µL fermentation medium in 24-well plates to an initial OD_600nm_ of 0.1. The 24-deep-well plates were cultivated at 30 °C, 800 rpm, and 90% moisture in INFORS Microtron (INFORS HT Multitron Pro, Switzerland). Cells of the stationary growth phase were used to detect the fluorescence intensity using a microplate reader (SpectraMax M5, Molecular Devices, USA, λ excitation = 560 nm, λ emission = 607 nm).

### l-Threonine production by cultivation in 24-deep-well plates

For fermentation in 24-well plates, the seed cultivation was performed in TSB medium, and the fermentation medium contains 80 g/L glucose, 1 g/L soya peptone, 1 g/L yeast extract, 1 g/L NaCl, 1 g/L (NH_4_)_2_SO_4_, 1 g/L K_2_HPO_4_·3H_2_O, 0.45 g/L MgSO_4_·7H_2_O, 0.05 g/L FeSO_4_·7H_2_O, 6 g/L urea, 400 µg/L biotin, 100 µg/L vitamin B1, and 40 g/L MOPS (pH 7.2). Kanamycin (25 µg/mL) and IPTG (0.5 mM) were added into medium as required. For seed preparation, strains were cultivated in 24-deep-well plates containing 800 µL TSB medium in each well. The preculture was used to inoculate 800 µL fermentation medium in 24-well plates to an initial OD_600nm_ of 0.1. The 24-deep-well plates were cultivated at 30 °C, 800 rpm, and 90% moisture in INFORS Microtron (INFORS HT Multitron Pro, Switzerland). During the cultivation process, no additional reagents were added to control pH. The urea and MPOS in the medium acted as pH buffers. Small amounts of samples were taken to detect pH during the anaphase of fermentation. To assess the maximum production potential of strains, the fermentation was terminated when the pH dropped to ∼ 5.5. After total cultivation of 24–36 h, samples were taken to analyze cell biomass and various amino acids.

### Fed-batch fermentation in a 5-L bioreactor

TSB medium was used for seed preparation. The fermentation medium contains 40 g/L glucose, 4 g/L soya peptone, 4 g/L yeast extract, 15 g/L (NH_4_)_2_SO_4_, 6 g/L KH_2_PO_4_, 0.5 g/L MgSO_4_·7H_2_O, 0.1 g/L FeSO_4_·7H_2_O, 0.05 g/L MnSO_4_·H_2_O, 4 g/L NaCl, 1 mg/L biotin, 1 mg/L vitamin B1, and suitable antifoam. Kanamycin (25 µg/mL) and IPTG (0.5 mM) were added into the initial fermentation medium. Fed-batch fermentations were conducted in a 5 L bioreactor (Bao xing Bio., Shanghai, China). Seeds were cultivated at 30 °C and 220 rpm for 8 h in a 500 mL shake flask containing 75 mL of TSB medium containing 25 µg/mL kanamycin. The seed culture was then transferred into a 5 L bioreactor containing 1.5 L fermentation medium with an inoculation volume of 10%. Temperature was maintained at 32 °C, and pH was automatically controlled at 7.0 by the addition of NH_4_OH. The dissolved oxygen was maintained at 30% by automatic adjustment of agitation and aeration rate. The sterile glucose solution (700 g/L) containing kanamycin (25 µg/mL) and IPTG (0.5 mM) was fed into the bioreactor at appropriate rates to maintain the glucose concentration in the range of 0–1 g/L. Samples at different time point were withdrawn from the bioreactor for measuring cell biomass, glucose, and various amino acids.

### Analytical methods

Cell density in 24-deep-well plates was monitored by measuring OD_600nm_ using Tecan Infinite® 200 Pro microplate reader (Tecan, China). Cell density in shake flask or bioreactors was monitored by measuring OD_600nm_ using an ultraviolet spectrophotometer (Shimadzu, Kyoto, Japan). Glucose in the medium was quantified using an SBA-40D biosensor analyzer (Institute of Biology of Shandong Province Academy of Sciences, Jinan, China) equipped with a glucose oxidase membrane. The concentrations of various amino acids were quantified using a HPLC method. Cell cultures were centrifuged at 12,000 rpm for 5 min, and the supernatant was used for determination after appropriate dilution. The HPLC system consists of a Prominence UFLC (Shimadzu, Japan) equipped with a Zorbax Eclipse AAA column (4.6 mm × 150 mm, 5 μm, Agilent Technologies, USA) and a UV detector (Wang et al. 2018). A gradient of 50 mM sodium acetate buffer at pH 6.4 with a gradient solution containing acetonitrile-water (50%, v/v) was utilized as the eluent. Various amino acids were detected as their 2,4-dinitrofluorobenzene derivatives at 360 nm by following the precolumn derivation method. Intracellular l-threonine was extracted using a centrifugal separation protocol involving centrifugation of cells through a layer of silicon oil (Forget et al. 1993), and then detected using the HPLC method described above. The intracellular volume used to calculate the intracellular l-threonine concentration was 1.7 µL/mg DCW (Ruffert et al. 1997).

**Abbreviations**.

**Table:** 

C.glutamicum	: Corynebacterium glutamicum
THF	: Tetrahydrofolate
Methyl THF	: 5,10-Methylene tetrahydrofolate
CDS	: 5′-End of coding sequences
RFP	: Red fluorescent protein
DAP	: d,l-Diaminopimelate
IC_50_	: Half maximal inhibitory concentration
AHV	: α-Amino-β-hydroxy-valeric acid
LB	: Luria-Bertani
IPTG	: Isopropyl-β-d-thiogalactopyranoside
Km	: Kanamycin

### Electronic supplementary material

Below is the link to the electronic supplementary material.

Supplementary Material 1

Supplementary Material 2

## Declarations

### Authorinformation

^1^State Key Laboratory of Biobased Material and Green Papermaking, Qilu University of Technology, Shandong Academy of Sciences, Jinan 250353, China. ^2^Key Laboratory of Engineering Biology for Low-carbon Manufacturing, Tianjin Institute of Industrial Biotechnology, Chinese Academy of Sciences, Tianjin, 300308, China. ^3^National Center of Technology Innovation for Synthetic Biology, Tianjin 300308, China. ^4^Shandong Provincial Key Laboratory of Microbial Engineering, School of Bioengineering, Qilu University of Technology, Shandong Academy of Sciences, Jinan 250353, China. ^5^Shandong University of Traditional Chinese Medicine, Jinan 250355, China.
