# Supplementary figures and images for: Reconstruction the feedback regulation of amino acid metabolism to develop a non-auxotrophic l-threonine producing Corynebacterium glutamicum
Source: Bioresour Bioprocess. 2024 Apr 26;11(1):43. doi: 10.1186/s40643-024-00753-9 (PMC11045695; doi:10.1186/s40643-024-00753-9)

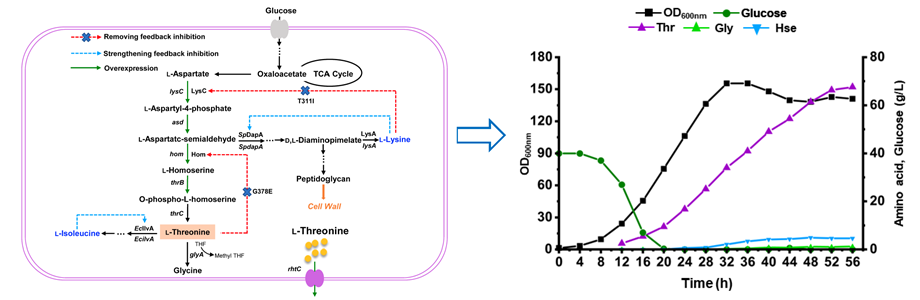

Supplement: Supplementary file 1 — Supplementary Material 1 [file 40643_2024_753_MOESM1_ESM.png]
