# Supplementary material for: Reconstruction the feedback regulation of amino acid metabolism to develop a non-auxotrophic l-threonine producing Corynebacterium glutamicum
Source: Bioresour Bioprocess. 2024 Apr 26;11(1):43. doi: 10.1186/s40643-024-00753-9 (PMC11045695; doi:10.1186/s40643-024-00753-9)
Supplement: Supplementary file 2 — Supplementary Material 2 [file 40643_2024_753_MOESM2_ESM.docx]

**Additional file**

**Reconstruction the feedback regulation of amino acid metabolism to develop a non-auxotrophic l-threonine producing *Corynebacterium glutamicum***

Jianhang Liu^1,2,3,4†^, Jiao Liu ^2,3†^, Jiajun Li^1,4^, Xiaojia Zhao^2,3^, Guannan Sun^2,3^, Qianqian Qiao^2,3^, Tuo Shi^2,3^, Bin Che^2,3^, Jiuzhou Chen^2,3^, Qianqian Zhuang^1,4,5^, Yu Wang^2,3^, Jibin Sun^2,3^, Deqiang Zhu^1,4*^, Ping Zheng^2,3*^

^1^State Key Laboratory of Biobased Material and Green Papermaking, Qilu University of Technology, Shandong Academy of Sciences, Jinan 250353, China

^2^Key Laboratory of Systems Microbial Biotechnology, Chinese Academy of Sciences, Tianjin Institute of Industrial Biotechnology, Tianjin 300308, China

^3^National Center of Technology Innovation for Synthetic Biology, Tianjin 300308, China.

^4^Shandong Provincial Key Laboratory of Microbial Engineering, School of Bioengineering, Qilu University of Technology, Shandong Academy of Sciences, Jinan 250353, China

^5^Shandong University of Traditional Chinese Medicine, Jinan 250355, China

^*^Correspondence: zheng_p@tib.cas.cn; zdq0819@qlu.edu.cn

^†^Jianhang Liu and Jiao Liu contributed equally to this work.


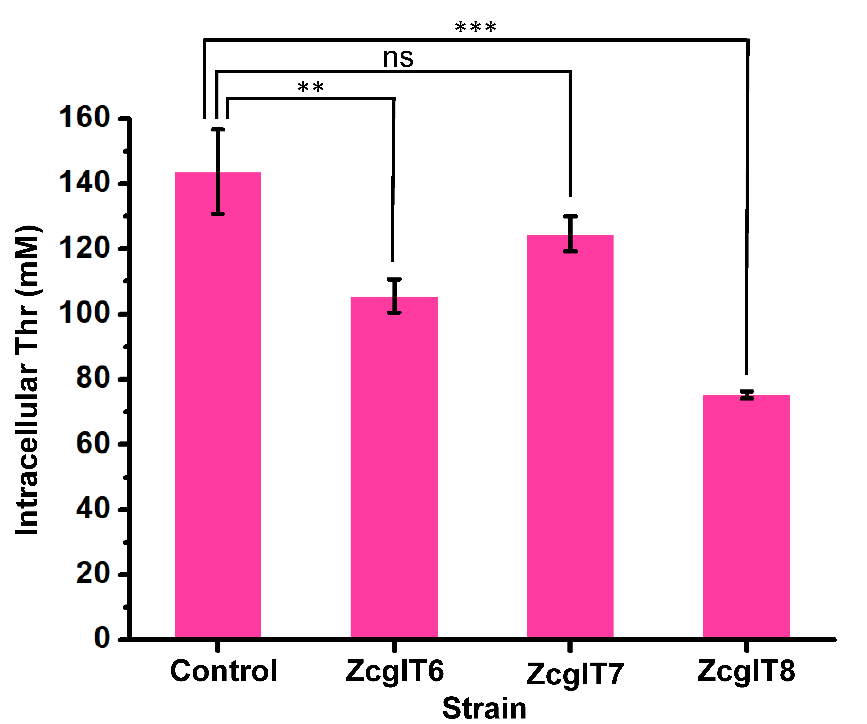


**Fig. S1** Intracellular l-threonine concentrations of strains with exporter overexpression*.* The 24-deep-well plate fermentation was performed at 30°C for 36 h with 0.5 mM IPTG. Enough cells were collected for the measurement of intracellular l-threonine. Control, ZcglT5 derivative harboring pEC-XK99E empty plasmid; ZcglT6, ZcglT5 derivative harboring pEC-*thrE* plasmid; ZcglT7, ZcglT5 derivative harboring pEC-*serE* plasmid; ZcglT8, ZcglT5 derivative harboring pEC-*rhtC* plasmid. Thr, l-threonine. Data are presented as mean values +/− SD (n=3 independent experiments). ns *P* < 0.05, ***P* < 0.01, ****P* < 0.001, student’s two-tailed *t*-test.

**Table S1.** Plasmids used in this study

| **Plasmid** | **Description^a^** | **Reference or source** |
| --- | --- | --- |
| pK18*mobsacB* | Suicide plasmid for genetic manipulation in *C. glutamicum*, Km^R^ | (Schäfer et al. 1994) |
| pEC-XK99E | Expression vector of *C. glutamicum*, IPTG-inducible promoter *P_trc_*, Km^R^ | (Kirchner et al. 2003) |
| pK18-*hom*^G378E^ | pK18*mobsacB* derivative carrying homology arms for G378E mutation of *hom* | This study |
| pK18-*lysC*^T311I^ | pK18*mobsacB* derivative carrying homology arms for T311I mutation of *lysC* | This study |
| pK18-*P_gpmA_*_-16_ | pK18*mobsacB* derivative carrying homology arms and *P_gpmA_*_-16_ promoter for *P_gpmA_*_-16_ promoter insertion | This study |
| pK18-*P_pyc_*_-20_ | pK18*mobsacB* derivative carrying homology arms and *P_pyc_*_-20_ promoter for *P_pyc_*_-20_ promoter insertion | This study |
| pK18-*EcilvA* | pK18*mobsacB* derivative carrying homology arms and *EcilvA* gene for *EcilvA* replacement | This study |
| pK18-*SpdapA* | pK18*mobsacB* derivative carrying homology arms and *SpdapA* gene for *SpdapA* replacement | This study |
| pEC-*thrE* | pEC-XK99E derivative carrying a *thrE* cassette driven by IPTG-inducible promoter *P_trc_* | This study |
| pEC-*serE* | pEC-XK99E derivative carrying a *serE* cassette driven by IPTG-inducible promoter *P_trc_* | This study |
| pEC-*rhtC* | pEC-XK99E derivative carrying a *rhtC* cassette driven by IPTG-inducible promoter *P_trc_* | This study |
| pEC-*rhtCthrB* | pEC-XK99E derivative carrying a *rhtC* cassette driven by IPTG-inducible promoter *P_trc_* and a *thrB* cassette driven by constitutive promoter H6 ^(Wei et al. 2018)^ | This study |
| pEC-*P_hom_*-*hom* | pEC-XK99E derivative carrying 180 bp of *hom* gene and *rfp* gene driven by constitutive promoter *P_hom_* | This study |
| pEC-*P_gpmA_*_-16_-*hom* | pEC-XK99E derivative carrying 180 bp of *hom* gene and *rfp* gene driven by constitutive promoter *P_gpmA_*_-16_ | This study |
| pEC-*P_pyc_*_-20_-*hom* | pEC-XK99E derivative carrying 180 bp of *hom* gene and *rfp* gene driven by constitutive promoter *P_pyc_*_-20_ | This study |
| pEC-*P_lysC_*-*lysC* | pEC-XK99E derivative carrying 180 bp of *lysC* gene and *rfp* gene driven by constitutive promoter *P_lysC_* | This study |
| pEC-*P_gpmA_*_-16_-*lysC* | pEC-XK99E derivative carrying 180 bp of *lysC* gene and *rfp* gene driven by constitutive promoter *P_gpmA-_*_16_ | This study |
| pEC-*P_pyc_*_-20_-*lysC* | pEC-XK99E derivative carrying 180 bp of *lysC* gene and *rfp* gene driven by constitutive promoter *P_pyc-_*_20_ | This study |

^a^Km^R^ represents resistance to kanamycin.

**Table S2.** Primers and details for constructing plasmids

| **Plasmid** | **Primer** | **Sequence 5’-3’** | **PCR template** | **Plasmid construction process^a^** |
| --- | --- | --- | --- | --- |
| pK18-*hom*^G378E^ | *hom*-B1 | AGCGGAAGAGCGCCCAATACCTCAGCAGTCGGAATTGCCC | Genomic DNA of *C. glutamicum* | Ligation of three PCR products via recombination |
|  | *hom*-B2 | ACGCGATCTTCCACATCCATG |  |  |
|  | *hom*-B3 | GGATGTGGAAGATCGCGTGGAGGTTTTG | Genomic DNA of *C. glutamicum* |  |
|  | *hom*-B4 | TGTAAAACGACGGCCAGTGCCCTTAATGCCAGACTCACGAGC |  |  |
|  | pK18-1 | GCACTGGCCGTCGTTTTAC | pK18*mobsacB* |  |
|  | pK18-2 | GTATTGGGCGCTCTTCCGCTTC |  |  |
| pK18-*lysC*^T311I^ | *lysC*-B1 | AGCGGAAGAGCGCCCAATACTCCTTAGGGAGCCATCTTTTGG | Genomic DNA of *C. glutamicum* | Ligation of three PCR products via recombination |
|  | *lysC*-B2 | GGTGATGTCGGTGGTGCCGTCTTCT |  |  |
|  | *lysC*-B3 | ACGGCACCACCGACATCACC | Genomic DNA of *C. glutamicum* |  |
|  | *lysC*-B4 | TGTAAAACGACGGCCAGTGCTTCATCGGTTTCGAAGGTGC |  |  |
|  | pK18-1 | GCACTGGCCGTCGTTTTAC | pK18*mobsacB* |  |
|  | pK18-2 | GTATTGGGCGCTCTTCCGCTTC |  |  |
| pK18-*P_gpmA-_*_16_ | *hom*-1 | AGCGGAAGAGCGCCCAATACTCCGTATGCAGTGAGCGTGGC | Genomic DNA of *C. glutamicum* | Ligation of four PCR products via recombination |
|  | *hom*-2 | CGCGGTCGAGCGTTGTTGTC |  |  |
|  | P*_gpmA-_*_16_-1 | GACAACAACGCTCGACCGCGCTGGGCCGACGCCTTAGC | P*_gpmA_*_-16_ variant plasmid of lab |  |
|  | P*_gpmA-_*_16_-2 | GGGGCAGATGCTGAGGTCATTTTTCCGTTAGTCATAGGTACTATCCTATCACAAAACTATTCAGGACGCGCGTTTACTTAGCCG |  |  |
|  | *hom*-3 | ATGACCTCAGCATCTGCCCCA | Genomic DNA of *C. glutamicum* |  |
|  | *hom*-4 | TGTAAAACGACGGCCAGTGCGCCCATCACAGACTGGATCTGATC |  |  |
|  | pK18-1 | GCACTGGCCGTCGTTTTAC | pK18*mobsacB* |  |
|  | pK18-2 | GTATTGGGCGCTCTTCCGCTTC |  |  |
| pK18-*P_pyc-_*_20_ | *lysC*-1 | AGCGGAAGAGCGCCCAATACCGTCACAAGACCAAGGATGAG | Genomic DNA of *C. glutamicum* | Ligation of four PCR products via recombination |
|  | *lysC*-2 | CTTTGTGCACCTTTCGATCTA |  |  |
|  | P*_pyc-20_*-1 | AGATCGAAAGGTGCACAAAGGAAAACCCAGGATTGCTTTGT | P*_pyc_*_-20_ variant plasmid of lab |  |
|  | P*_pyc-20_*-2 | TAGAGTAATTATTCCTTTCAA |  |  |
|  | *lysC*-3 | TGAAAGGAATAATTACTCTAATGGCCCTGGTCGTACAGAAAT | Genomic DNA of *C. glutamicum* |  |
|  | *lysC*-4 | TGTAAAACGACGGCCAGTGCGGAGCCAACAGCAGCAAGTTCC |  |  |
|  | pK18-1 | GCACTGGCCGTCGTTTTAC | pK18*mobsacB* |  |
|  | pK18-2 | GTATTGGGCGCTCTTCCGCTTC |  |  |
| pK18-*EcilvA* | *ilvA*-1 | ACATAGCTGAAGGCCACCTCAAT | Genomic DNA of *C. glutamicum* | Ligation of four PCR products via recombination |
|  | *ilvA*-2 | TGTAAAACGACGGCCAGTGCTGATTCAGATGAAGTCCGCAATTAT |  |  |
|  | *EcilvA*-1 | GAAGATTACACTAGTCAACC ATGGCTGACTCGCAACCCCT | Genomic DNA of *E. coli* |  |
|  | *EcilvA*-2 | GAGGTGGCCTTCAGCTATGT CTAACCCGCCAAAAAGAACCTGA |  |  |
|  | *ilvA*-3 | AGCGGAAGAGCGCCCAATACTCTGTTATCTACCCTGGTGTTG | Genomic DNA of *C. glutamicum* |  |
|  | *ilvA*-4 | GGTTGACTAGTGTAATCTTCTC |  |  |
|  | pK18-1 | GCACTGGCCGTCGTTTTAC | pK18*mobsacB* |  |
|  | pK18-2 | GTATTGGGCGCTCTTCCGCTTC |  |  |
| pK18-*SpdapA* | *dapA*-1 | AGCGGAAGAGCGCCCAATACGGAGCGTTTAAAGGCTGTGGCCGA | Genomic DNA of *C. glutamicum* | Ligation of four PCR products via recombination |
|  | *dapA*-2 | AGAGTTCAAGGTTACCTTCTTC |  |  |
|  | *SpdapA*-1 | AGAAGGTAACCTTGAACTCTATGTCTTATCAAGATTTAAAAGA | Synthetic *dapA* gene from *Streptococcus pneumoniae* |  |
|  | *SpdapA*-2 | GTAATCTGGTCTTAAGACCCCTGT |  |  |
|  | *dapA*-3 | GGGTCTTAAGACCAGATTACCCGAAAAAAGCTGGAGTTCTATAAATATGAATGATTCCCGAAATCGC | Genomic DNA of *C. glutamicum* |  |
|  | *dapA*-4 | TGTAAAACGACGGCCAGTGCGGGACAATGCAGGCAGGTCAG |  |  |
|  | pK18-1 | GCACTGGCCGTCGTTTTAC | pK18*mobsacB* |  |
|  | pK18-2 | GTATTGGGCGCTCTTCCGCTTC |  |  |
| pEC-*thrE* | *thrE*-1 | ACAGGCCAAAGGAGTTGAGAATGTTGAGTTTTGCGACCCT | Genomic DNA of *C. glutamicum* | Ligation of two PCR products via recombination |
|  | *thrE*-2 | CCAAGCTTGCATGCCTGCAGTTACCTTTTATTACCGAATC |  |  |
|  | pEC-1 | CTGCAGGCATGCAAGCTTG | pEC-XK99E |  |
|  | pEC-2 | TCTCAACTCCTTTGGCCTGTG |  |  |
| pEC-*serE* | *serE*-1 | ACAGGCCAAAGGAGTTGAGATTGGCAATAATCAAGGGCAT | Genomic DNA of *C. glutamicum* | Ligation of two PCR products via recombination |
|  | *serE*-2 | CCAAGCTTGCATGCCTGCAGTTAACTAGGTGTGTGTACTC |  |  |
|  | pEC-1 | CTGCAGGCATGCAAGCTTG | pEC-XK99E |  |
|  | pEC-2 | TCTCAACTCCTTTGGCCTGTG |  |  |
| pEC-*rhtC* | *rhtC*-1 | ACAGGCCAAAGGAGTTGAGAATGTTGATGTTATTTCTCACCGT | Genomic DNA of *E. coli* | Ligation of two PCR products via recombination |
|  | *rhtC*-2 | CCAAGCTTGCATGCCTGCAGTCACCGCGAAATAATCAAAT |  |  |
|  | pEC-1 | CTGCAGGCATGCAAGCTTG | pEC-XK99E |  |
|  | pEC-2 | TCTCAACTCCTTTGGCCTGTG |  |  |
| pEC-*rhtCthrB* | *thrB*-1 | TTCTTGACGAGTTCTTCTGAGCTCCCGGCGCTACATATATTTCGACCTTTCTGACGGAATTGGAATGTGTTATACTATACTTGACTGTATCGTAAAGGAAGGAGATGGCAATTGAACTGAACGTCG | Genomic DNA of *C. glutamicum* | Ligation of two PCR products via recombination |
|  | *thrB*-2 | CGCGAACCCCAGAGTCCCGCTAAGGTTCACGTGTTCAGCGAATAA |  |  |
|  | pEC-3 | GCGGGACTCTGGGGTTCGC | pEC-*rhtC* |  |
|  | pEC-4 | TCAGAAGAACTCGTCAAGAAGGCGA |  |  |
| pEC-*P_hom_-hom* | P*_hom_*-1 | CCTGATGCGGTATTTTCTCC GTCCTCCCCGGGTTGATATTA | Genomic DNA of *C. glutamicum* | Ligation of three PCR products via recombination |
|  | P*_hom_*-2 | GATTCTCCAAAAATAATCGCGG |  |  |
|  | *hom*180-1 | GCGATTATTTTTGGAGAATCATGACCTCAGCATCTGCCCC | Genomic DNA of *C. glutamicum* |  |
|  | *hom*180-2 | CCACCTCCAGAGCCACCGCCAGAAACAGCAATGCCACGAACC |  |  |
|  | pEC-5 | GGCGGTGGCTCTGGAGGTGGT | pEC-1 ^(Liu et al. 2022)^ |  |
|  | pEC-6 | GGAGAAAATACCGCATCAGGC |  |  |
| pEC-*P_gpmA-_*_16_*-hom* | P*_gpmA_*_16_-1-1 | CCTGATGCGGTATTTTCTCCCTGGGCCGACGCCTTAGCCA | pK18-P*_gpmA_*_-16_ | Ligation of two PCR products via recombination |
|  | P*_gpmA_*_16_-1-1 | ATAGTACCTATGACTAACGGAAAAATGACCTCAGCATCTGCCCC |  |  |
|  | pEC-7 | ATGACCTCAGCATCTGCCCCAAG | pEC-P*hom*-*hom* |  |
|  | pEC-6 | GGAGAAAATACCGCATCAGGC |  |  |
| pEC-*P_pyc-20_*-*hom* | P*_pyc-20_*-1-1 | CCTGATGCGGTATTTTCTCCGAAAACCCAGGATTGCTTTGTG | pK18-P*_pyc-_*_20_ | Ligation of two PCR products via recombination |
|  | P*_pyc-20_*-2-1 | GGGGCAGATGCTGAGGTCATTAGAGTAATTATTCCTTTCAACA |  |  |
|  | pEC-7 | ATGACCTCAGCATCTGCCCCAAG | pEC-P*hom*-hom |  |
|  | pEC-6 | GGAGAAAATACCGCATCAGGC |  |  |
| pEC-*P_lysC_-lysC* | P*_lysC_*-1 | CCTGATGCGGTATTTTCTCC TTGATTCAGGGTAGTTGACTAAAGA | Genomic DNA of *C. glutamicum* | Ligation of three PCR products via recombination |
|  | P*_lysC_*-2 | TTCTGTACGACCAGGGCCACCTTTGTGCACCTTTCGATCTA |  |  |
|  | *lysC*180-1 | GTGGCCCTGGTCGTACAGAA | Genomic DNA of *C. glutamicum* |  |
|  | *lysC*180-2 | CCACCTCCAGAGCCACCGCCAACGGGATTCACTGCCGCTGCAAGT |  |  |
|  | pEC-5 | GGCGGTGGCTCTGGAGGTGGT | pEC-P*hom*-hom |  |
|  | pEC-6 | GGAGAAAATACCGCATCAGGC |  |  |
| pEC-*P_gpmA-16_-lysC* | P*_gpmA_*_16_-1-1 | CCTGATGCGGTATTTTCTCCCTGGGCCGACGCCTTAGCCA | pK18-*P_gpmA_*_-16_ | Ligation of two PCR products via recombination |
|  | P*_gpmA_*_16_-1-2 | TTCTGTACGACCAGGGCCATTTTTCCGTTAGTCATAGGTAC |  |  |
|  | pEC-9 | ATGGCCCTGGTCGTACAGAAATA | pEC-*P_lysC_*-*lysC* |  |
|  | pEC-6 | GGAGAAAATACCGCATCAGGC |  |  |
| pEC-*P_pyc-20_*-*lysC* | P*_pyc-20_*-1-1 | CCTGATGCGGTATTTTCTCCGAAAACCCAGGATTGCTTTGTG | pK18-P*_pyc-_*_20_ | Ligation of two PCR products via recombination |
|  | P*_pyc-20_*-2-2 | TTCTGTACGACCAGGGCCATTAGAGTAATTATTCCTTTCAAC |  |  |
|  | pEC-9 | ATGGCCCTGGTCGTACAGAAATA | pEC-*P_lysC_*-*lysC* |  |
|  | pEC-6 | GGAGAAAATACCGCATCAGGC |  |  |

**Table S3.** Promoters used in this study

| **Promoter** | **Sequence** |
| --- | --- |
| *P_gpmA_*_-16_ | CTGGGCCGACGCCTTAGCCACACTCTTGGACGATGACGAAACGCGCATCAGAATGGGTGAAGACGCCGTCGAACACGCCAGAACATTCTCCTGGGCGGCCACCGCCGCACAGCTATCGTCGCTGTACAACGACGCTATTGCCAACGAAAATGTCGACGGTGAAACGCATCACGGCTAAGTAAACGCGCGTCCTGAATAGTTTTGTGATAGGATAGTACCTATGACTAACGGAAAA |
| *P_pyc_*_-20_ | GAAAACCCAGGATTGCTTTGTGCACTCCTGGGTTTTCACTTTGTTAAGCAGTTTTGGGGAAAAGTGCAAAGTTTGCAAAGTTTAGAAATATTTTAAGAGGTAAGATGTCTGCAGGTGGAAGCGTTTAAATGCGTTAAACTTGGCCAAATGTGGCAACCTTTGCAAGGTGAAAAACTGGGGCGGGGTTAGATCCTGGGGGGTTTATTTCATTCACTTTGGCTTGAAGTCGTGCAGGTCAGGGGAGTGTTGCCCGAAAACATTGAGAGGAAAACAAAAACCGGGCCTTGATTGTAAGATAAGACATTTAGTATAATTAGACGCAGTGACTGCTATCACCCTTGGCGGTCTCTTGTTGAAAGGAATAATTACTCTA |

**Supplementary references**

Kirchner O, Tauch A (2003) Tools for genetic engineering in the amino acid-producing bacterium *Corynebacterium glutamicum*. J Biotechnol 104:287-299

Liu J, Liu M, Shi T, Sun G, Gao N, Zhao X, Guo X, Ni X, Yuan Q, Feng J, Liu Z, Guo Y, Chen J, Wang Y, Zheng P, Sun J (2022) CRISPR-assisted rational flux-tuning and arrayed CRISPRi screening of an l-proline exporter for l-proline hyperproduction. Nat Commun 13:891

Schäfer A, Tauch A, Jäger W, Kalinowski J, Thierbach G, Pühler A (1994) Small mobilizable multi-purpose cloning vectors derived from the *Escherichia coli* plasmids pK18 and pK19: selection of defined deletions in the chromosome of *Corynebacterium glutamicum*. Gene 145:69-73

Wei L, Xu N, Wang Y, Zhou W, Han G, Ma Y, Liu J (2018) Promoter library-based module combination (PLMC) technology for optimization of threonine biosynthesis in *Corynebacterium glutamicum*. Appl Microbiol Biotechnol 102:4117-4130
